# Jaundice outbreak likely caused by HEV in Amritsar, Punjab, India, 2013

**DOI:** 10.1186/s12889-019-6786-1

**Published:** 2019-05-10

**Authors:** Tripurari Kumar, Aakash Shrivastava, Deepak Bhatia, Yash Mitra, Anil Kumar, Sharmeen Hussain, Lakhbir Singh Chauhan, Kayla F. Laserson, Jai Prakash Narain, Rajesh Kumar, Averhoff Francisco

**Affiliations:** 1 National Centre for Disease Control, 22-Sham Nath Marg, Delhi, 110054 India; 2 Department of Health and Family Welfare, Chandigarh, Punjab India; 3 Emory University, Atlanta, GA USA; 4 India-Centers for Disease Control and Prevention, Delhi, India; 5 Post Graduate Institute of Medical Education & Research, Chandigarh, India; 6 Centers for Disease Control and Prevention, Atlanta, USA

**Keywords:** Amritsar, Hepatitis E, Integrated disease surveillance program, Global Health security, Outbreak, Punjab, Risk factors

## Abstract

**Background:**

Hepatitis-E Virus (HEV) infection is endemic in Punjab, India. On 4th April 2013, public officials of Labour Colony, Amritsar reported > 20 jaundice cases occurring within several days.

**Methods:**

We performed a case-control study to identify the cause and prevent additional cases of jaundice cases in Amritsar, Punjab, India in 2013.

**Results:**

A total of 159 cases (attack rate 3.6%) and 1 death were identified in Labour and 5 adjoining colonies from January 1 to June 5, 2013. Persons with jaundice were more likely to report foul-smelling piped water (adjusted odds ratio [AOR], 4.0, 95% confidence interval [CI], 2.2–7.2) and used piped water for drinking (AOR, 5.1; 95% CI, 2.2–11.4) than persons without jaundice. Among 14 cases tested, all had anti-hepatitis E virus IgM, and none had anti-hepatitis A virus IgM. Additionally, 21/23 tap water samples from affected households had detectable fecal coliforms. An environmental investigation found that water pipelines were damaged during sewer construction and likely led to contamination of drinking water with hepatitis E virus.

**Conclusions:**

Hepatitis E outbreaks are common in India, to curb future outbreaks of hepatitis E; measures to ensure safe drinking water are urgently needed**.**

## Article summary

An investigation into a 2013 outbreak of jaundice in Amritsar, India found the likely cause was hepatitis E virus from a contaminated water pipeline broken during sewer construction, an accident residents were not alerted to.

## Introduction

There are 20 million Hepatitis E virus (HEV) infections that result in 44,000 deaths annually as per World Health Organization (WHO) estimates [1, 2]. More than 60% of recognized HEV infections and deaths occur in South Asia [3]. Although HEV is endemic is India and outbreaks are regularly reported [4], field investigations of HEV outbreaks are infrequent, limiting opportunities to describe the epidemiology, risk factors, and interventions for prevention. Rapid detection and control of HEV outbreaks and other public health threats at their source is goal of implementation of the WHO International Health Regulations and are essential for global health security.

Amritsar is a large city in the state of Punjab in the northern part of India and situated next to the Pakistan border. Amritsar has a population of 2.5 million [5] and the city has grown quickly with insufficient infrastructure for water and sanitation. Also within Amritsar is the Golden Temple, a famous Sikh shrine, which has more than 100,000 visitors per day [6]. The area of the city reporting the outbreak has a large population of migrant settlers with poor infrastructure.

The IDSP collects weekly surveillance data on food- and water-borne forms of viral hepatitis A and E along with 18 other epidemic-prone diseases. All government-run health centers and hospitals and a few designated private facilities serve as reporting units (RUs) to the IDSP. District Surveillance Units (DSUs) receive weekly surveillance reports of viral hepatitis cases and outbreaks from the RUs and report weekly to IDSP surveillance offices via a web portal (www.idsp.nic.in) [4]. The DSUs investigate and report on suspected hepatitis outbreaks, defined as > 2 clinical cases of acute jaundice per week in their jurisdiction and submit findings to IDSP. Twelve outbreaks of hepatitis E has been reported in 2012 across various districts of Punjab, India.

On April 4, 2013, a public representative of Labour Colony, Amritsar informed a DSU that > 20 jaundice cases had occurred in Labour and 5 adjoining colonies, which had been noticed over the last few weeks. The population of the 6 affected colonies is approximately 4440. Residents are supplied piped water 3 times a day via 4 interconnected tubewells. We performed an investigation of the jaundice outbreak and a case-control analysis to determine the etiological agent, source of the infection, risk factors for infection and means to prevent further transmission of HEV in this outbreak and opportunities to prevent future outbreaks.

## Materials and methods

### Investigation and case-control analysis

We conducted a 1:1 case-control study of all cases to identify the etiologic agent and risk factors associated with the jaundice outbreak in the 6 colonies. A line list of clinical cases was prepared by conducting a door-to-door search on 5th & 6th April 2013. A jaundice case patient was defined as a person who resided in Labour Colony, Dhakka Colony, Baba Jeevan Singh Colony, Sat Kartar Nagar, Vikas Nagar or Shakti Nagar and reported jaundice with at least 1 of the following symptoms — anorexia, nausea, vomiting, abdominal pain, or fever between December 1, 2012 and July 15, 2013. Jaundice is defined as yellowish discoloration of the skin, the conjunctival membranes over the sclerae (whites of the eyes) and other mucous membranes caused by elevated blood bilirubin levels. Controls were defined as having no history of jaundice in the previous 6 months. Controls were selected from the nearest unaffected household of each case household until the required numbers of controls were recruited. The control could be any age and was the first person who responded at the household. One of the case patients died was excluded from the study.

Cases and controls were administered a questionnaire enquiring about risk factors and potential exposures for infection. Demographic information including age, sex, education, family size, religion and specific location of the home was also collected. Potential risk factors included history of foul-smelling water, source of household water, water storage and purification methods and hand washing practices.

### Investigation of environmental factors

We inspected sources of drinking water (tubewells, municipal water supply pipelines and private borewells) and sewage drainage systems. Tap water specimens were collected from case as well as control houses at point when outbreak was at its peak by convenience sampling. We enquired about mass gatherings (e.g. social gatherings, weddings in the community) and about construction activity that occurred in the prior 6 months. Additionally, we interviewed agencies involved in development of new sewer lines in the area to ascertain if there were recent changes in water supply systems.

### Laboratory evaluations

Serum samples from case patients were tested for antibodies to hepatitis E virus (HEV, anti-HEV IgM) and hepatitis A virus (anti-HAV IgM) by enzyme-linked immunosorbent assay (ELISA). For tap water specimens, the most probable number (MPN) method was used for coliform enumeration. All assays were conducted by the Microbiology Department, Government Medical College, Amritsar.

### Data analyses

The collected data were entered into Epi Info-7 for analysis. We performed bivariate and multivariate analysis to calculate odd ratios (OR), adjusted OR (AOR) and 95% confidence interval (CI) for risk factors associated with the outbreak. For the multivariate logistic regression, we used a backwards elimination modeling strategy and evaluated all variables that were statistically significant (*p* < 0.05) in bivariate analyses.

## Results

### Description of the outbreak

**Fig. 1:**
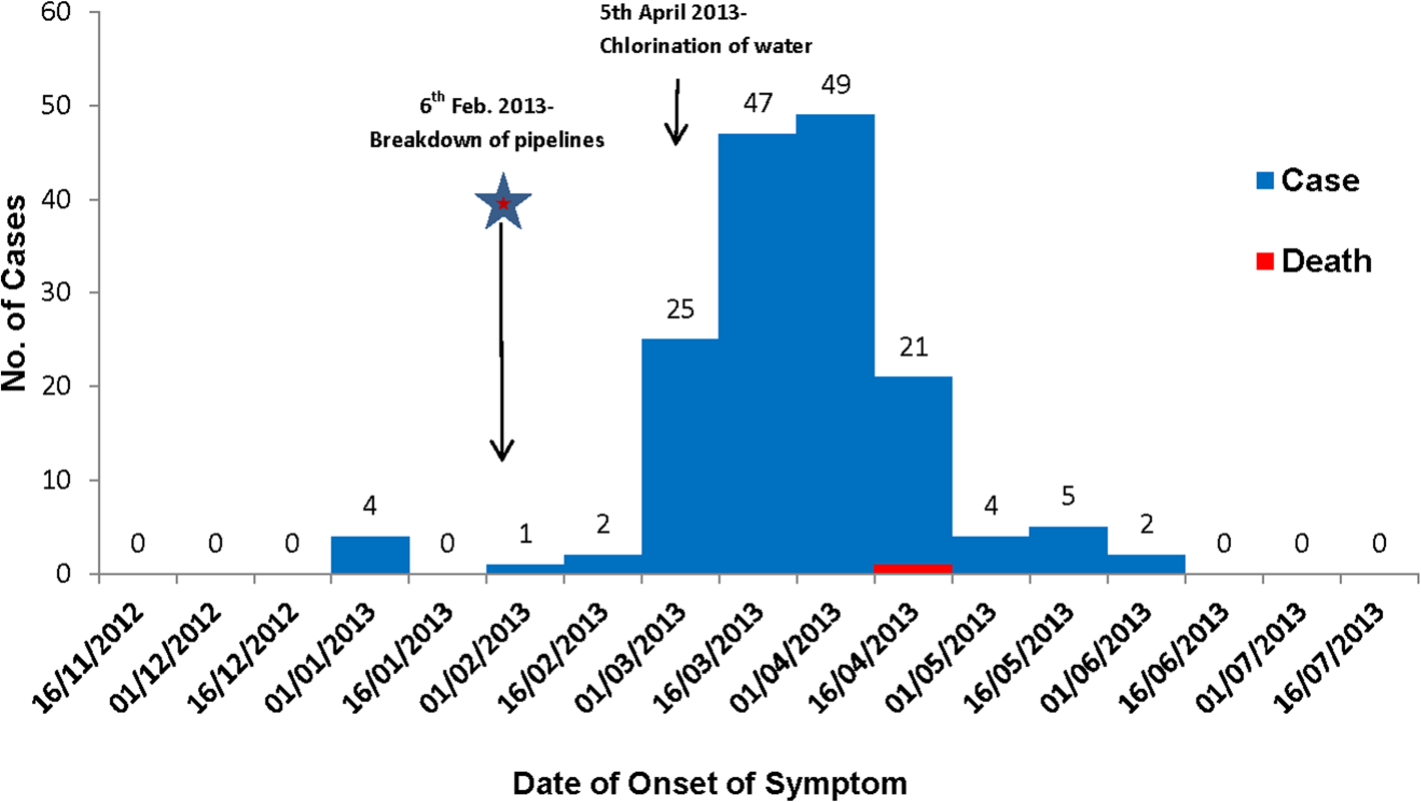
Epidemic Curve of Jaundice Cases (*N* = 160), Amritsar, Punjab, India, January to June 2013

We identified 160 persons who fit the case definition (community attack rate [AR], 3.6%). Onset was January 1, 2013 for the first known case and June 5, 2013 for the last case (Fig. 1). 159 cases were included in our analyses. One of the case patients died was excluded from CCS; the patient was a 72-year-old female with history of hypertension and diabetes excluded. No pregnant women were reported as case patients. Plotting the onset of illness indicated a point source epidemic with a median incubation period of 53 days assuming 6th February 2013 as the potential date of exposure.

**Fig. 2:**
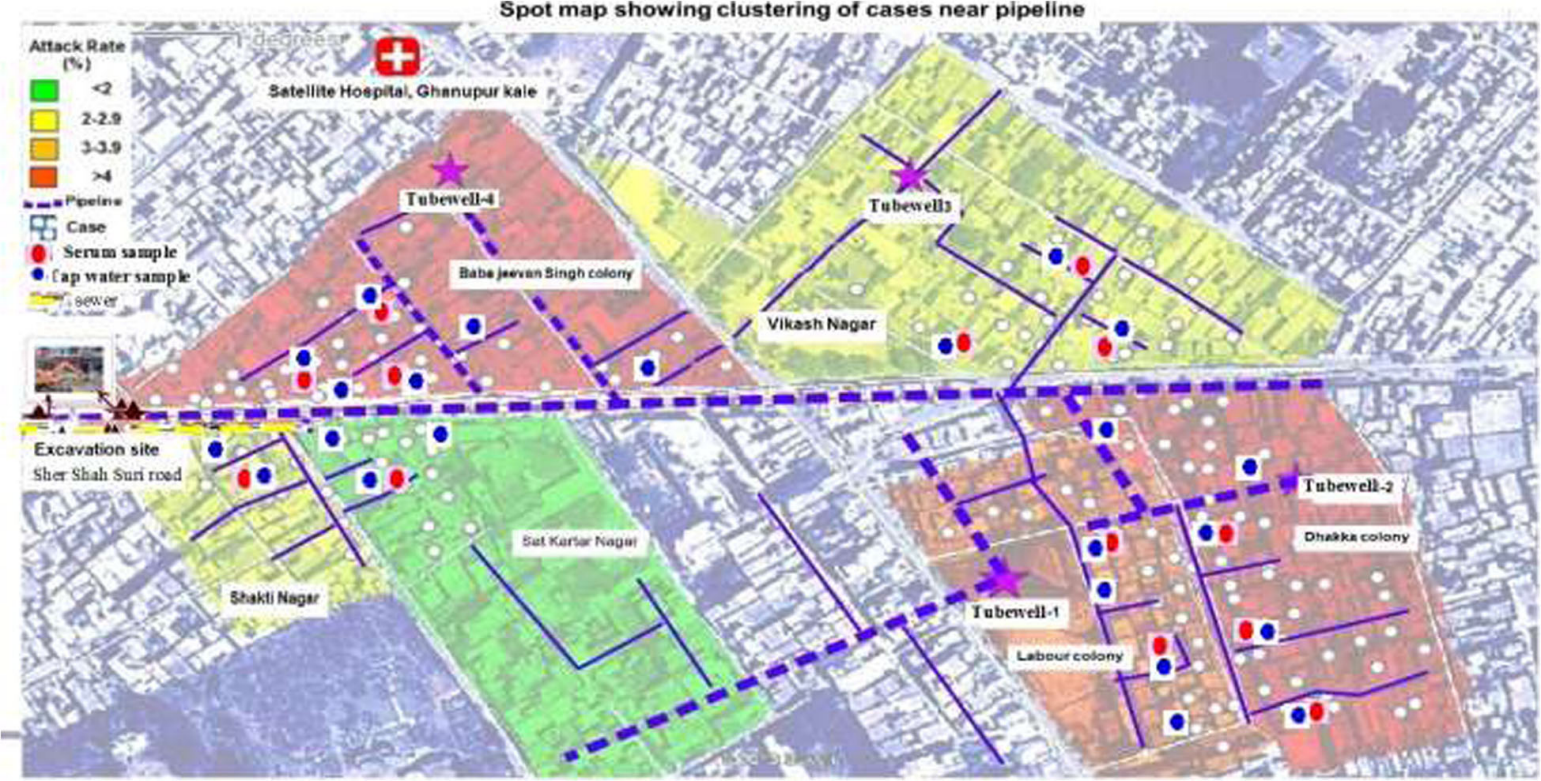
Map of Jaundice Cases (*N* = 160) by Attack Rates and Pipelines, Amritsar, Punjab, India, January–June 2013

All 6 colonies were affected. Attack rates varied with Dhakka Colony experiencing the highest (6.3%), followed by Baba Jeevan Singh Colony (4.4%) and Labour Colony (4.0%) (Table 1; Fig. 2). Males accounted for 56% of the cases (AR, 3.8%; Table 2) and the median age of case patients was 33 years (range 7–75 years). Persons aged 15–34 years accounted for 54.7% of cases and had the highest AR (4.7%).

**Table 1:** Jaundice Attack Rate by Age Sex and Locality, Amritsar, Punjab, January–June 2013

Characteristics	Population	Cases	Attack Rate (%)
Age groups (years)			
0–4	360	0	0
5–14	792	12	1.5
15–34	1857	87	4.7
35–59	1037	44	4.2
> 60	394	16	4.1
Sex			
Male	2314	89	3.8
Female	2126	70	3.3
Locality			
Dhaka Colony	789	50	6.3
Baba Jeevan Singh Colony	724	32	4.4
Labour Colony	527	21	4
Shakti Nagar	448	12	2.7
Vikas Nagar	1301	32	2.5
Sat Kartar Nagar	651	12	1.8
Total	4440	159	3.6

**Table 2:** Jaundice Attack Rate by Age and Sex, Amritsar, Punjab, January–June 2013

Characteristics	Population	Cases	Attack Rate (%)
Age groups (years)			
0–4	360	0	0
5–14	792	12	1.5
15–34	1857	87	4.7
35–59	1037	44	4.2
> 60	394	16	4.1
Sex			
Male	2314	89	3.8
Female	2126	70	3.3
Total	4440	159	3.6

No mass gatherings or other community events were reported to occur during or preceding the outbreak. Mapping revealed a clustering of cases around a new sewer line construction site and a damaged water/sewer pipeline (Fig. 2). A total of 14 samples (5 samples collected on 5th April, 6 samples on 6th April and 3 samples on 16th April) were collected on convenience and sent to microbiology department, Govt. Medical College, Amritsar. All 14 were positive for anti-HEV IgM and negative for anti-HAV IgM.

### Risk factors

Overall, 159 cases and 159 controls were included in the case-control study (Table 3). The median age of case was 33 years (range 7–75) and control was 34 years (range 9–75). On bivariate analyses, cases were significantly more likely to have a family size ≥5 (OR, 1.5; 95% CI, 1.0–2.4), to be non-Sikh (OR, 1.9; 95% CI, 1.2–3.0), to complain of foul smelling water (OR, 2.8; 95% CI, 1.8–4.5), and to use piped water for drinking (OR, 4.7; 95% CI, 2.5–8.7). Boiling water before drinking showed a protective effect (OR 0.1; 95% CI: 0.1–0.3). Income, food habits, toilet use, and hand washing practices were not significantly associated with having jaundice.

**Table 3:** Risk Factors Associated With Jaundice, Amritsar, Punjab, India, 2013

Factors	Cases		Controls		OR	95% CI	AOR	95% CI
	*N* = 159	%	*N* = 159	%				
Male	89	56	91	57.2	0.9	0.6–1.4		
Illiterate	14	8.8	17	10.7	0.8	0.4–1.7		
Family size ≥5	81	51	63	39.6	1.6	1.0–2.4	1.6	0.9–2.7
Non-Sikh (Immigrants)	106	66.6	81	50.9	1.9	1.2–3.0	1.7	0.9–2.8
Water foul smelling	106	66.7	65	40.9	2.8	1.8–4.5	4.0	2.2–7.2
Pipe water supply vs. bore wells	143	89.9	104	65.4	4.7	2.5–8.7	5.1	2.2–11.4
Bucket vs. overhead tank water storage	100	62.9	59	37.1	2.8	1.8–4.5	1.9	0.9–3.5
Purification method vs. none								
Chlorine	41	25.8	19	11.9	1.2	0.6–2.4	0.5	0.2–1.0
Boiling	17	10.7	55	34.6	0.1	0.1–0.3	0.1	0.05–0.2
Filter	47	29.6	54	34	0.5	0.2–0.9	1.5	0.6–3.4
Hand washing after defecation with soap vs. not washing	144	90.6	147	87	1.4	0.7–2.8		
Hand washing with soap before eating	122	76.7	130	76.9	0.9	0.5–1.6		

After controlling for religion, family size and water storage in house, a multivariate analysis indicated foul-smelling water (AOR, 4.0; 95% CI 2.2–7.2), drinking piped water (AOR, 5.1; 95% CI, 2.2–11.4), and boiling water before drinking (AOR, 0.09; 95% CI, 0.05–0.2) remained significantly associated with jaundice (Table 3).

### Water investigation

The main source of drinking water was through tubewell. Tubewell is basically vertical drilled wells, bored into an underground aquifer in the earth’s surface, to extract water for various purposes (domestic, peri-domestic or farming). A chlorinator is advised in the tubewell so chlorinated supply of water reaches to house. The water can be used for drinking after chlorination. There were 4 tubewells (T1–4, Fig. 2), which were interconnected to each other so that if one tubewell is working can supply water to area where tubewell is not working. The Tubewell at the colony supplied water for 3 intervals each day (8–10 am, 12–2 pm, and 7–9 pm). The tubewell in Dhakka colony (T3: Fig. 2) was non-functional from October 2012 to April 2013.

Chlorination of water supplies was irregular and unreliable; only 2 of the 4 tubewells had functioning chlorination systems. Of the 23 tap water samples (19 from case houses and 4 from control houses) collected on convenience. 14 tap-water samples were from same houses where serum samples were collected and sent to Public Health Lab for testing. 21 samples (19 cases plus 2 controls) were contaminated with fecal coliforms (median 44 CFU/100 ml).

The construction of a new sewer line being laid in Sher Shah Suri road, intersecting Baba Jiwan Singh Colony and Sat Kartar Nagar (Fig. 2), began October 12, 2012 and continued during the outbreak. Officials who were responsible for digging and putting in new sewer lines, had broken the older sewer pipe & drinking pipelines during excavation confirmed that damage had occurred and indicated it resulted in contamination. Further, the log book of the executing agency also confirmed that pipelines were damaged on October 28, 2012, January 16, 2013, and February 6, 2013. They repaired the first two instances of damage quickly but delayed the last occurrence.

## Discussion

This outbreak of jaundice in 6 colonies in Amritsar, Punjab India was likely the result of contamination of the municipal drinking water supply with HEV. Amritsar City lacks a sewage treatment plant (STP) resulting in untreated wastewater being discharged into the surrounding rivers [7]. To rectify this situation, the city received funding support for a project to set up STPs and lay sewer lines from the Japan Bank for International Cooperation (JBIC). The municipal drinking water pipeline was reportedly damaged during excavator digging for new sewer line. The outbreak was associated with a third event associated with this infrastructure project that resulted in compromised municipal drinking water; the first 2 instances were small and repaired quickly, but in this third instance, not only was the repair delayed, but the local residents were also not informed of the break or advised to stop drinking the pipeline water. The breach in drinking water supply pipelines likely resulted in contamination of the drinking water and this outbreak. Repair work on water distribution systems can present a risk for contamination of drinking water if guidelines are not followed, such as having documented protocols, adopting general precautionary working practices, using health criteria to select personnel, implementing effective procedures for cleaning and disinfection, assessing the risks and monitoring the effects of both planned and emergency engineering work [8]. Standard guidelines of municipal corporations in India suggest that during construction projects that involve excavation, construction agencies should seek coordination with relevant departments, inclusive of the area’s water supply department, such coordination may have averted this outbreak and could prevent similar outbreaks in the future.

Our results indicated a point source epidemic with a median incubation period of 53 days. Persons most affected in this analysis were between 15 and 34 years of age. This is consistent with prior hepatitis E outbreaks in India, where since 1956 the peak incidence of disease has been among persons aged 15–30 years [9–11]. In Nepal, 75% of symptomatic cases of HEV infection in epidemic situations have occurred among persons aged 15–34 years [12]. Clinical observations in Bangladesh [13] and Pakistan [14] have also documented that the frequency of overt HEV disease is highest among those aged 21–40 years and lowest among those younger than the age of 10 years.

HEV is an important cause of morbidity and mortality in India, where the predominant strain of HEV is genotype 1 [15]. It is estimated that 2 million cases of hepatitis E occur annually in India [16, 17]. Hepatitis E is endemic in Punjab state [18], where in 2012, 12 outbreaks were reported from different districts through the IDSP [4, IDSP Punjab data]. Outbreaks of hepatitis E are most commonly associated with heavy rainfall and disruption of water supplies [17, 19]. Spread mainly through fecal contamination of water supplies or food [20, 21], hepatitis E is usually self-limiting and does not result in long-term sequelae, although occasionally fulminant hepatitis develops, with 3.3 million acute cases annually worldwide [22]. HEV infection during pregnancy can take a severe course, with up to 20% fatality rates [20, 22]; reasons for this are not known. There is no evidence hepatitis E infection results in either chronic inflammation or an asymptomatic chronic carrier state [22].

The findings of our study suggested that people who drank piped water with a foul smell during the outbreak period had a greater risk of illness. Many outbreaks of HEV infection reported in India have been attributed to sewage contamination in broken water pipelines [23–25].

The authorities repaired the damaged water pipelines at the excavation site when they received information from the residents complaining of foul smelling and dirty water from the drinking pipelines; they also chlorinated the water. However, chlorination alone may be unsuccessful in controlling a hepatitis E outbreak [26], and therefore all drinking water should have been boiled or bottled from a safe source. At a later date, the water supply department cut the water supply in area where construction of the new sewer line was occurring and provided an alternate water supply (via a water tanker) to the area. Further, the health department also instructed people to drink boiled water. The health department also requested that the interconnection between the tube wells be disconnected and to follow WHO safety guidelines during new construction and repair of pipelines [8]. Health educational activities in the community regarding personal and domestic hygiene were also provided through mass media and consumption of safe water was stressed.

Our study has several limitations. First, cases were self-identified and subject to ascertainment or recall bias. Second, we could not obtain an existing map of the water pipelines of Amritsar from the Municipal Corporation. Third, due to constrain of resources we could not lab test for each & every cases. Fourth, we did not evaluate levels of aminotransferases and therefore may have missed anicteric cases. Fifth, we did not laboratory confirm all icteric cases, so we might have misclassified some as hepatitis E. Finally, we could not test controls for HEV antibodies, which could have biased our findings if some controls were actually cases.

## Conclusion

Our findings suggest that the hepatitis outbreak in early 2013 in Amristar, Punjab, India was most likely caused by contamination of the municipal water supply with HEV. The contamination occurred due to a break in the municipal water pipes during sewer construction. To prevent future outbreaks, authorities were alerted to the need to follow guidelines during construction and repair of pipelines. This field investigation demonstrates that rapid detection and response to HEV outbreaks can lead to important control efforts and prevention of future outbreaks. Such efforts are essential for ensuring global health security.
